# Root of Tooth in the Antrum of Highmore

**Published:** 1867-06

**Authors:** Gam’l Jackson


### ROOT OF TOOTH IN THE ANTRUM OF HIGHMORE.

BY GAM'L JACKSON.

TOMES, in his Dental Surgery, speaks of an accident which may happen in extracting teeth, and relates a case which Dr. Cattlin had. It was my misfortune to add another to the list, under the following circumstances:

In July last a lady called at my office to have the left superior first molar extracted. The crown had decayed away leaving the roots but slightly connected. I removed one of the buccal roots with the forceps, but failing with this instrument with both the other roots, I resorted to the straight elevator. The remaining buccal root was thus readily removed, but in attempting to dislodge the palatine root my first effort resulted in pushing it into the antrum.

Not having suitable instruments at hand for retrieving the accident, I appointed the next morning for the operation. My patient kept her engagement, but would neither be narcotized nor submit without. I dismissed her with instructions to return upon the appearance of certain symptoms. These set in at the expiration of twenty days, and my patient returned with a fetid purulent discharge from the left nostril, and was quite willing that I should operate. I enlarged the socket through which the root entered with a four-line trocar, but exhausted my skill, and with ever variety of probe, in trying to find the root, I could find nothing but the tubers corresponding to the root of the adjacent teeth. Topid water was thrown through the opening and the probes tried again, but with no better success.

The case was now postponed with the prophetic remark from the attending physician, who chloroformed the patient, that "the root would probably fall through the opening in a short time."

The Doctor's prophecy came to pass. The lady was dining the next day on potatoes, and a portion crowded past the lip of a rubber plate, which partly covered the opening. An effort to draw out the potatoe with the tongue, brought the root rattling against the plate!

The root was three-eights of an inch long and just the right shape to favor its passage into the antrum; slightly enlarged at the point and regularly tapered to the other extremity. Had it not been discharged within a few days it was my intention to remove the second bicuspid, and after enlarging its socket with a smaller trocar than the one used in the first instance, to cut out the intervening bone. The opening thus made would have enabled me to find the root with certainty. Before cutting out the bone between the two perforations, however, a strong current of water thrown through the socket of the bicuspid would probably have washed the root out through the first opening. I have always considered elevators cruel and inefficient instruments, and have never had the least success with any but the straight one, described on page 126 of Robinson, on Extracting Teeth, and called the "Gouge." This may sometimes be used to advantage in removing the roots of the six front teeth of the upper jaw. Badly decayed roots of this class may be thus removed with perhaps less pain and less mutilation of the surrounding tissues than would attend the use of the bone forceps—a result particularly desirable where artificial teeth are to be made, and plain teeth are indicated. But my general practice is to use Parmley's bone forceps for all kinds of roots, whether their crowns have been broken off or lost by decay. I consider these instruments the most indispensable of any in my extracting case.

